# Investigating functional brain networks in multiple sclerosis patients: a graph theory approach for evaluating working memory impairment

**DOI:** 10.1186/s40708-026-00324-y

**Published:** 2026-07-26

**Authors:** Sareh Yousefi, Mohammad Reza Daliri

**Affiliations:** Neuroscience & Neuroengineering Research Lab., Biomedical Engineering Department, School of Electrical Engineering, Iran University of Science & Technology (IUST), Narmak, Tehran, Iran

**Keywords:** Working memory, fMRI, Multiple sclerosis, Graph theory, Functional connectivity

## Abstract

This study introduces a graph theory-based machine learning framework to analyze functional brain networks in Multiple Sclerosis (MS) patients during working memory tasks. Using fMRI data from 22 participants (8 MS patients/14 controls) performing a Persian n-back task, we constructed functional connectivity networks with an automated anatomical labeling atlas (116 regions). Novel methodological contributions include: a proportional thresholding approach (5–80% connectivity strength) to optimize network analysis, and extraction of six graph-theoretic features (degree, clustering coefficient, betweenness/eigenvector/page-rank/k-coreness centrality) for classification. Machine learning models (SVM, decision trees, kNN) were trained on task-specific networks (1-back, 2-back, 3-back), with leave-one-subject-out validation. The 3-back task (high cognitive load) yielded superior classification (95.5% accuracy) using SVM with degree, k-coreness, and eigenvector centrality features, outperforming traditional resting-state approaches. Key innovations include identification of the cerebellum (1-back) and orbitofrontal cortex (3-back) as novel discriminative hubs, and demonstration that task difficulty modulates network separability. Results indicate MS patients exhibit impaired high-load connectivity patterns, with the proposed framework showing potential as an auxiliary diagnostic tool. The study provides both a technical blueprint for task-based network analysis and clinically relevant insights into MS-related cognitive impairment, bridging engineering methodologies with neurological applications.

## Introduction

Multiple sclerosis (MS) is an autoimmune disease of the central nervous system (CNS) and, compared with other neurological diseases, primarily affects young adults [1]. Myelin is a protective sheath that surrounds neuronal axons and allows rapid transmission of electrical impulses. This insulating sheath is produced by oligodendrocytes, a group of neuron-protective cells. In MS, oligodendrocytes are attacked by the immune system, leading to demyelination and the formation of plaques or sclerosis within the CNS [2]. These pathological changes disrupt effective axonal transmission, and as a result, MS patients exhibit sensory, motor, and cognitive impairments. More than 50% of MS patients experience cognitive impairment during the course of the disease [3], including deficits in attention, information processing speed, episodic memory, and executive functions [4–9]. Cognitive impairments can appear at early stages of MS and may progress over time; therefore, early detection and intervention are essential [10].

Among cognitive impairments, working memory (WM) dysfunction is particularly prominent in MS patients. WM refers to the brain^’^s ability to temporarily retain and actively manipulate information [11]. Deficits in maintaining task-relevant information may lead to slow or inconsistent processing and interfere with everyday activities. Pathological studies suggest that white matter damage plays a key role in WM dysfunction [12, 13].

Functional magnetic resonance imaging (fMRI) is a non-invasive technique that indirectly reflects neuronal activity by measuring blood-oxygen-level-dependent (BOLD) signals [14]. Task-based and resting-state fMRI are widely used to investigate brain activation and functional connectivity. MRI/fMRI provides a suitable framework for examining MS-related lesions and alterations in brain connectivity and has been extensively applied to detect changes in functional and structural brain architecture [15]. Studies indicate that, beyond focal tissue loss, cognitive impairment in MS may be associated with a cumulative failure of the brain^’^s adaptive capacity, resulting in abnormal activation patterns during cognitive and motor tasks [16, 17].

The human brain operates as a complex network of interacting elements with dynamic structural and functional connections. Graph-theoretical analysis provides a framework for characterizing large-scale brain networks and their topological properties. Disruptions in network organization may contribute to neurocognitive deficits in MS.

In this study, we investigate working memory–related functional brain network alterations in MS patients using task-based fMRI and graph-theoretical measures. The proposed approach aims to identify network-level markers associated with WM dysfunction and improve the understanding of cognitive impairment in MS. Details of the participants, task, data acquisition, and analysis pipeline are provided in the Methods section.

## Related work

### Working memory assessment using the n-back task

The n-back task is one of the most commonly used paradigms for exploring working memory. Many functional neuroimaging studies have employed this task to investigate brain activation associated with WM. For example, a high-cognitive verbal n-back task has been used to examine brain activation in early-stage Parkinson^’^s disease [18], while other studies investigated functional connectivity in adolescents with epilepsy. The n-back task has also been applied in cognitive disorders such as schizophrenia [19] and depression [20]. In MS, Rocca et al. [17] conducted a multicenter study comparing functional correlations during n-back task performance between MS patients and healthy controls, showing that preserved frontal lobe activity was associated with better cognitive performance.

### fMRI and graph-theoretical analysis in MS

The human brain is a complex network of interacting elements with dynamic structural and functional connections. Graph theory is widely used to study these networks, particularly functional connectivity.

Disruptions in neural networks may lead to neurocognitive deficits. Several studies have applied graph-theoretical analysis to explore these abnormalities in MS patients [17, 21–24]. Some studies report that MS patients exhibit increased prefrontal cortex activity compared to healthy controls during WM tasks [25]. Resting-state fMRI studies show increased functional connectivity in sensory-motor and cognitive networks [26], and enhanced connections between the default mode network, prefrontal network, and other brain networks in cognitively impaired MS patients [27].

### Machine learning approaches in MS

Machine learning (ML) techniques are increasingly used to distinguish patients from healthy controls based on brain network features. Several studies have successfully classified MS patients using task-based or resting-state fMRI data [23, 28–30]. For example, Ashtiani et al. [23] differentiated MS patients using graph features extracted from task-related fMRI during the Paced Auditory Serial Addition Task (PASAT), achieving 81.25% sensitivity with a support vector machine (SVM) classifier. Saccà et al. [29] applied different classifiers to resting-state fMRI data, identifying the sensorimotor network as the most discriminative and achieving 85.7% accuracy with Random Forest and SVM. Beyond MS, Grueso et al. [31] reviewed ML applications for predicting Alzheimer^’^s disease in individuals with mild cognitive impairment. Although these methods require further development, they have significant potential for clinical applications.

## Methods

**Fig. 1:**
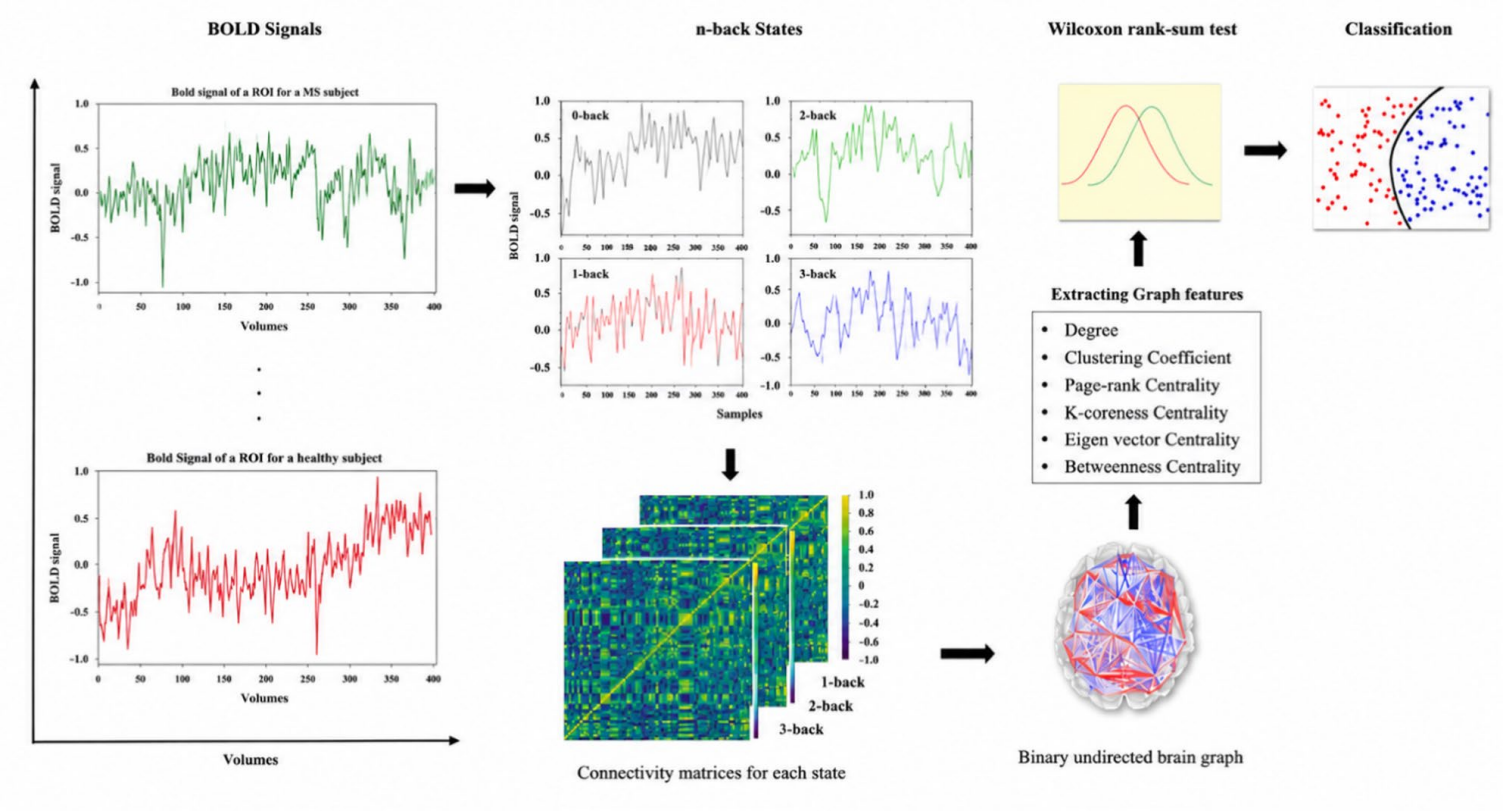
The schematic diagram of the proposed method

Figure 1 illustrates the overall workflow of the proposed methodology. The following subsections describe the participant characteristics, data acquisition, stimulation paradigm, and analysis procedures.

### Participants

Twenty-two participants, including eight patients with MS and fourteen healthy controls (HC), were recruited for this experiment. The MS patients included seven females and one male (right-handed) with a mean age and standard deviation of 34.75 ± 6.036. They had been diagnosed with the disease for under 96 months and had an average disease diagnosis time of 32.5 months with varied attack numbers. The healthy group included nine females and five males (right-handed) with a mean age and standard deviation of 30.57 ± 4.67. All patients were diagnosed with relapsing–remitting MS (RRMS) by a neurologist using the McDonald criteria [32].

All experimental procedures were carried out according to the institutional and/or national research committee^’^s ethical standards, as well as the 1964 Helsinki declaration and its subsequent amendments or comparable ethical standards. The experimental protocols were also approved by Neuroscience & Neuroengineering Research Laboratory ethic committee of Iran University of Science & Technology (IUST). Informed consent was obtained from all subjects taking part in the experiment.

### Stimulation paradigm

The n-back task is a neurocognitive task that examines working memory at various cognitive loads [33]. In this study, fMRI data were acquired while each participant performed the visual Persian version of the n-back task. The task presented sequences of the Persian alphabet on the screen in relation to n-back conditions. The task included four levels of difficulty. In the 0-back task, participants were asked to respond when the target letter “ژ” appeared on the screen. This condition was considered as a control state and was not used in the analysis. We employed 1-, 2- and 3-back tasks as the task conditions. In the 1-back task, if a letter was repeated after a random letter, the subjects were required to press the response key. Likewise, in the 2-back and 3-back tasks, if a letter was displayed again after two and three different letters the subjects were required to press the response key, respectively.

**Fig. 2:**
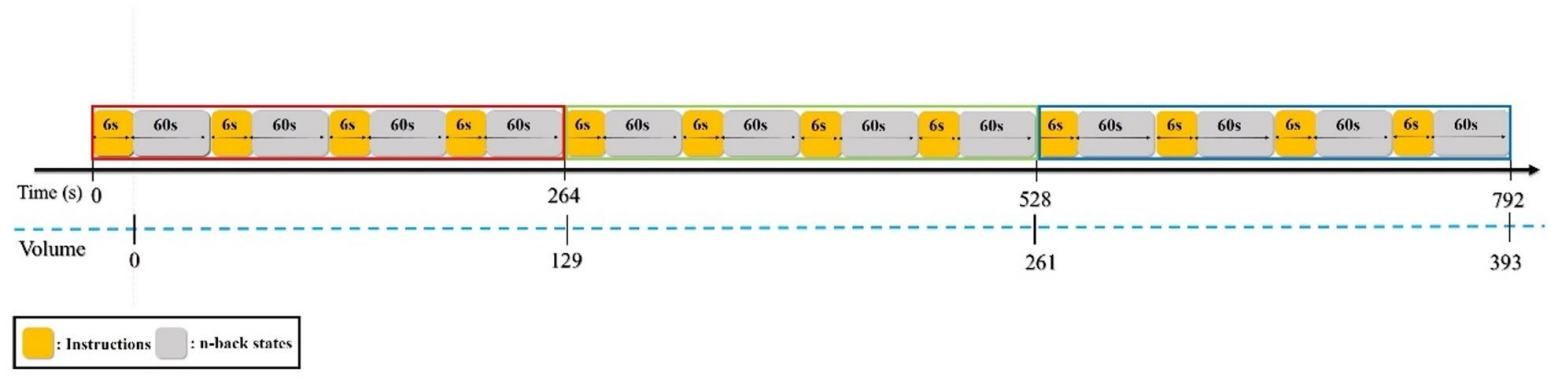
Block diagram of the designed n-back task

There were three sessions, which consisted of four blocks, and each block appeared once in random order in each session. Each block lasted 60 s, and before each session began, the subject was instructed how to perform the task that took 6 s as shown in Fig. 2.

### Data acquisition

A 3.0 T Siemens Tim Trio MRI scanner was used to collect both structural and functional MRI data. For structural images, a high-resolution three-dimensional T1-weighted MPRAGE pulse sequence protocol was employed. The parameters for this protocol were as follows: repetition time (TR) of 1.80 s, echo time (TE) of 3.44 ms, flip angle of 7 degrees, matrix size of 256 × 256, voxel size of 1 × 1 × 1 mm^3^, and 176 sagittal slices per volume. Functional imaging, on the other hand, was performed using the echo-planar imaging (EPI) sequence. The parameters for the functional imaging protocol were as follows: TR of 2 s, TE of 30 ms, flip angle of 90 degrees, matrix size of 64 × 64, voxel size of 3 × 3 × 4 mm^3^, and 30 slices per volume. Each functional MR image consisted of a 4-dimensional dataset with dimensions of 64 × 64 × 30 × 396, where the first three dimensions represented a brain volume, and the fourth dimension represented the number of time samples.

### Pre-processing

The fMRI data were analyzed using SPM8 on MATLAB. The first three volumes collected were discarded from the analysis (as shown in Fig. 1) in order to eliminate the effect of magnetic moments. After converting raw DICOM images to NIFTI format, temporal and spatial pre-processing was performed. The processing stage consisted of slice timing correction, motion correction, high-pass temporal filtering (cut-off = 264), normalization to MNI coordinates, and spatial smoothing using a Gaussian filter (5 mm full-width-half maximum).

### Functional brain network construction

Functional connectivity refers to statistical dependency between different brain regions. To construct functional brain networks, automated anatomical labeling (AAL) atlas was applied [34]. Accordingly, in the present study, the whole brain of each participant was divided into 116 regions of interest (ROI). Then, the average time series of voxels within each region was calculated and standardized using z-score normalization prior to functional connectivity estimation. Following this, the n-back tasks that occurred randomly within each trial were separated and grouped together based on their onset time. That is to say, in each trial, there were 30 volumes related to each condition and by considering three trials we had a 90 volumes signal for each of the n-back tasks. Then, the brain regions were represented by nodes, and the Pearson correlation coefficients within nodes were considered as edges in the brain graph. For each participant, binary functional connectivity were obtained corresponding to each task. As a result, four 116 by 116 matrices were earned. These obtained matrices were fully connected and these dense networks increase the computational cost. In addition, these connections contain redundant information. To address this issue, proportional thresholding (PTh) was employed which preserves a fraction of the strongest connections [30]. To construct thresholded matrices, PTh values were used by preserving 5–80 percent of the strongest connections with a step size of 5%. Then, connections in which the weight was less than the obtained threshold were converted to 0, while those exceeding the threshold were converted to 1. As a result, binary, unweighted and undirected matrices were acquired.

Functional connectivity networks were constructed using Pearson correlation followed by proportional thresholding and binarization. This approach was chosen to reduce the influence of inter-subject variability in overall connectivity strength and to facilitate comparison with previous graph-theoretical studies in MS. However, binarization inevitably discards information about connection weights, which may limit sensitivity to subtle connectivity differences. Future studies could extend this framework by incorporating weighted or directed graphs, as well as effective connectivity approaches, to capture more detailed network dynamics.

Proportional thresholding was applied over a wide density range in 5% increments to ensure network connectivity and to avoid bias associated with a single arbitrary threshold. Lower densities may lead to fragmented networks, whereas higher densities approach fully connected graphs and reduce topological specificity. The selected range and step size are consistent with prior graph-theoretical studies and allow assessment of the robustness of network measures across multiple thresholds.

**Fig. 3:**
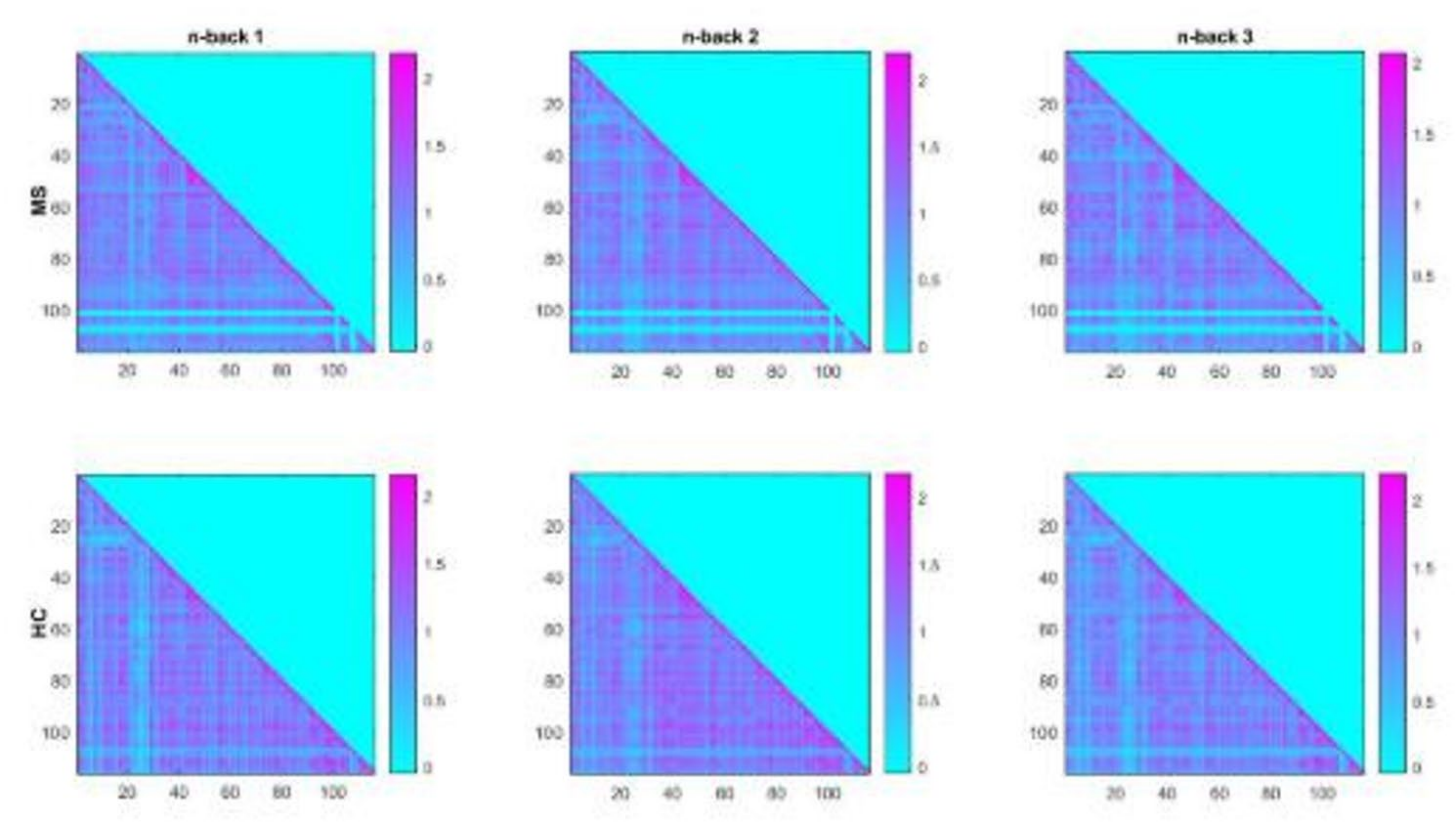
Group-averaged functional connectivity matrices for MS patients and healthy controls across n-back conditions. Matrices show Fisher z-transformed Pearson correlations with a common color scale; only the lower triangle is displayed

Figure 3 illustrates the group-averaged functional connectivity matrices for MS patients and healthy controls across three n-back conditions. All matrices represent Fisher z-transformed Pearson correlation coefficients and are displayed using a common color scale, with only the lower triangle part shown to avoid redundancy.

Visual inspection of the connectivity matrices reveals condition-dependent changes in both groups. In healthy controls, increasing working memory load from n-back 1 to n-back 3 is associated with more homogeneous and structured connectivity patterns, suggesting a progressive engagement of large-scale brain networks. In contrast, MS patients exhibit more heterogeneous connectivity patterns across conditions, with pronounced variability at higher n-back levels.

Notably, MS patients show localized increases in connectivity strength across specific regions, particularly under higher working memory load, whereas healthy controls display more evenly distributed connectivity patterns. These observations suggest alterations in functional network organization in MS during working memory processing.

Graph theoretical metrics, such as degree, clustering coefficient, betweenness centrality, page-rank centrality, eigenvector centrality, and k-correness centrality, were computed using the Brain Connectivity Toolbox [35, 36]. Degree reveals the number of edges connected to a single node. A node with a higher degree serves as a communication hub in the network of brain regions. The fraction of triangles around a single node is known as the clustering coefficient and is equivalent to the fraction of neighbors of the node that are neighbors of each other. It is a measure of separation and illustrates the tendency of nodes to create connected neighbors [37]. Betweenness centrality is defined as the fraction of the shortest paths within the network that pass through a given node. Furthermore, it describes a node^’^s effect on information flow within the network. Bridging nodes that connect disjoint parts of the network often have higher betweenness centrality [38]. Page rank centrality is a type of eigenvector centrality defined as the stationary distribution of a random process whose states are the nodes of the graph [39]. Eigenvector centrality examines the quantity and quality of the connections of a node and is determined based on the eigenvalues and eigenvectors of the adjacency matrix [40]. A high eigenvector score means that a node is connected to many nodes who themselves have high scores. K-coreness centrality is defined as a network core that is a set of nodes that are mutually and densely connected. In a binary network, k-core is the largest subgraph that contains nodes with minimum degree k [41]. The k-coreness centrality of a node is k if the node belongs to the k-core but not to the (k + 1)-core. The formula of the local features are represented in Table 1.

**Table 1:** The formula of graph local measures

Graph Measure	Formula
Degree	K_i_ $=\sum_{j\epsilon N}\mathrm{a}\mathrm{i}\mathrm{j}$
Local clustering coefficient	$$\mathrm{C}\mathrm{i}=\left\{\begin{array}{c}\frac{2\mathrm{t}\mathrm{i}}{ki(ki-1)} ki\ge 2 \\ 0 ki<2\end{array}\right.$$
Average clustering coefficient	$C= \frac{1}{n}\sum_{i\epsilon N}\mathrm{C}\mathrm{i}$
Betweenness centrality	B_i_ = $$\frac{1}{(n-1)(n-2)}\sum_{\begin{array}{c}h,j\epsilon N\\ h\ne j,h\ne i,j\ne i\end{array}}\frac{\upsigma \mathrm{h}\mathrm{j}(\mathrm{i})}{\upsigma \mathrm{h}\mathrm{j}}$$
Page-rank centrality	PR = α*P* **PR** + (1-α)e C_P_(i) = *PR*_*i*_ Where *P* is the transition probability matrix, α is the damping factor (typically α = 0.85), and *PR*_*i*_ denotes the PageRank centrality of node i e is a vector with equal entries
Eigenvector centrality	C_E_(i) = v_i_ Where i is the i-th element of the dominant eigenvector v of the network^’^s adjacency matrix
k-correness centrality	C_k_ (i)$=\mathrm{max}\left\{k \right|i$ ∈ k-core}

N is the set of all nodes in the network. a_ij_ is defined as the connection between node i and j. t_i_ is the number of triangles around a node i

Given that all extracted features were local, for each local feature, the dimension of feature vector was equal to 116 according to 116 brain regions. It is worth mentioning that features were extracted separately for each of the four constructed networks. Furthermore, these local features were extracted per participant at PTh values.

### Feature selection and classification

The purpose of feature selection is to select a small subset of the feature set by removing redundant and irrelevant features, which ultimately leads to a better model and less computational cost. The number of features compared to the number of subjects can cause the model to overfit. Multiple scalar feature selection methods were applied, for instance, mutual information and Fisher score, but it seemed as if the non-parametric Wilcoxon rank sum test performed better than others. In this study, to reach optimal performance, the number of features was increased from 1 to 10 features per threshold, and the results related to the optimal threshold and optimal number of features in each threshold have been reported in the next section.

In order to distinguish between the MS group and healthy controls, we applied different classification algorithms. These included support vector machine (SVM), decision trees (DTS), and k-nearest neighbors (kNN). By applying these different algorithms, we were able to evaluate the performance of each method and determine which was the most effective in distinguishing between the two groups. SVMs have been extensively utilized to identify patients with neurocognitive disorders [23, 28, 42–44]. In addition, it is a robust algorithm when we face a limited number of samples. In this study, linear, polynomial, and RBF kernels were implemented. One of the simplest classifiers is the k-nearest-neighbors. It is a non-parametric method that performs based on a similarity metric, which is the distance between the test set and the train set. In this case, kNN algorithm was developed with a k value of 5, which represented the square root of the number of total samples, and the similarity metric was chosen as Euclidean distance. The decision tree is a non-parametric approach that partitions the dataset according to specific criteria designed to minimize errors b at the terminal nodes, or leaves. To evaluate the classification performance, a leave-one-subject-out (LOSO) cross-validation strategy was employed, in which one subject was held out as the test sample while all remaining subjects were used for model development, and this procedure was repeated until each subject had served once as the test case [45]. All graph-theoretical features were initially extracted for every subject; however, feature selection was performed separately within each LOSO fold to prevent information leakage. Specifically, for each iteration: (1) the dataset was divided into a training set (N − 1 subjects) and one independent test subject; (2) feature ranking was performed exclusively on the training set using the Wilcoxon rank-sum test; (3) the top-ranked features (ranging from 1 to 10 features for each threshold) were selected based solely on the training data; (4) the selected feature subset was then applied to both the training data and the held-out test subject; (5) the classifier was trained using only the reduced training set; and (6) the trained model was evaluated on the unseen test subject. This entire pipeline, including feature ranking, feature selection, model training, and testing, was repeated independently for every LOSO iteration, ensuring that no information from the test subject was used during feature selection or model training.

All analyses were implemented in MATLAB R2019b (MathWorks, Natick, MA, USA) using the Statistics and Machine Learning Toolbox (Version 11.6).

Due to the exploratory nature of this study and the limited sample size, no explicit correction for multiple comparisons was applied at this stage.

In this work, the analysis was restricted to local graph-theoretical measures in order to focus on region-specific connectivity alterations associated with multiple sclerosis during working memory processing. Local metrics are particularly suitable for capturing focal network disruptions and allow straightforward regional interpretation, while global measures provide coarse summaries of whole-brain organization and may exhibit limited sensitivity in small and imbalanced samples. Limiting the feature set to local measures also helped control model complexity and reduce the risk of overfitting.

Similarly, classification was limited to SVM, kNN, and Decision Tree models, which are commonly applied in neuroimaging studies with small datasets due to their robustness and relatively low model complexity. The evaluation of more complex or highly parameterized classifiers was beyond the scope of the present study and is left for future investigations with larger cohorts.

We used accuracy, sensitivity, specificity, Cohen^’^s kappa statistics, and F1-score to evaluate our models. Accuracy is defined as the number of correct predictions to total predictions. In addition to accuracy, sensitivity, and specificity are usually mentioned. The sensitivity measure refers to the fraction of MS patients who were distinguished correctly from the total patients, while specificity shows the corresponding fraction for HCs.

F1-score is defined as the harmonic mean between precision and recall, the better F1-score represents better model performance.

In addition, Cohen^’^s kappa statistic is a measure for assessing the quality of model prediction, which is essential to use for imbalanced class problems.

## Results

The objective of this study is to differentiate between MS patients and healthy controls based on a modified version of the visual n-back task commonly used to assess working memory. Averaged BOLD signals for each ROI extracted and after separating volumes related to each n-back condition, the adjacency matrices were obtained by calculating correlation coefficient between brain regions. To mitigate the possibility of overfitting, we employed the leave-one-subject-out cross validation method, which involves evaluating each subject only once. Then, local features at various PThs for all constructed networks were extracted and the Wilcoxon rank sum test was utilized to determine the optimal number of features. Two groups were classified using multiple classifiers, including SVM, kNN, and DTs. Finally, the results have been reported based on the best performance for each n-back task and the optimal number of features and PThs.

Compared to local features, global features were found to be less effective, while local features were able to discriminate between MS patients and healthy controls. The classification accuracy by utilizing local features are presented in Table 2. This table constitutes from local features including the clustering coefficient, page-rank centrality, k-coreness centrality, eigenvector centrality, degree and betweenness centrality. Besides these features, the table also includes the performance metrics obtained from the three supervised learning algorithms used in this study. The results of the study revealed that the 3-back network was the most informative in differentiating between MS patients and healthy controls based on the selected local features. Specifically, this network exhibited the highest discriminative power, achieving an accuracy of over 80% and an f1-score of over 0.7 in classifying the two groups. What is more, the results generally suggest that the 2-back network exhibited suboptimal performance and lacked discriminative features compared to the other two networks. It is noteworthy that the 1-back network exhibited comparable discriminative power to the 3-back network in certain features, despite differences in the optimal features and thresholds at which these results were obtained. Moreover, the selected local graph features (degree, k-coreness centrality, and eigenvector centrality) were associated with the highest classification performance, achieving an accuracy of 95.5% and an F1-score above 0.9 for the 3-back condition within the studied cohort.

**Table 2:** Classification performances of the optimal sets of features in different cognitive loads

Graph measures	n-back network	Classifier	PTh (%)	Accuracy (%) ± SD	Sensitivity (%)	Specificity (%)	F1 Score	Number Of features	Cohen^’^s kappa	ROI
Clustering coefficient	3-back	SVM(polynomial)	40	**81.8% ± 0.395**	**87.5%**	**78.57%**	0.77	3	0.62	*Amygdala_L* *Postcentral_L*
	2-back	Decision Tree	20	77.3% ± 0.429	62.5%	85.7%	0.66	5	0.49	*Cerebellum_7b_L* *Lingual_R*
	1-back	Decision Tree	55	**81.8% ± 0.395**	**87.5%**	**78.57%**	0.77	2	0.62	*Cerebellum_3_R* *Temporal_Sup_L*
Page-rank centrality	3-back	Decision Tree	35	**86.4% ± 0.351**	**75%**	**92.86%**	**0.81**	2	0.69	*Frontal_Mid_Orb_L* *Heschl_R*
	2-back	All	50	81.8% ± 0.395	75%	85.71%	0.75	1	0.60	*Postcentral_L*
	1-back	SVM (Linear)	5	**86.4% ± 0.351**	**75%**	**92.86%**	**0.8**	6	0.69	*Postcentral_L* *Cerebellum_6_L* *Cerebellum_7b_L* *Cerebellum_9_R*
k-coreness centrality	3-back	KNN	75	**95.5% ± 0.213**	**100%**	**92.86%**	**0.94**	2	0.90	*Cuneus_R* *Heschl_R*
	2-back	Decision Tree	40	77.3% ± 0.429	87.5%	71.43%	0.73	2	0.54	*Rolandic_Oper_R* *Olfactory_L*
	1-back	Decision Tree	50	81.8% ± 0.395	75%	85.71%	0.75	1	0.60	*Supp_Motor_Area_R*
Eigenvector centrality	3-back	SVM(Linear/polynomial)	30–35	**95.5% ± 0.213**	**87.5%**	**100%**	**0.93**	2	0.89	*Frontal_Mid_Orb_L* *Heschl_R*
	2-back	Decision Tree	45	59.1% ± 0.503	75%	50%	0.57	4	0.22	*Frontal_Inf_Orb_R* *Olfactory_L* *Frontal_Sup_Orb_L*
	1-back	Decision Tree	60	81.8% ± 0.395	87.5%	78.57%	0.77	4	0.62	*Temporal_Pole_Sup_L* *Olfactory_L* *SupraMarginal_L*
Degree	3-back	Decision Tree	35	**95.5% ± 0.213**	**87.5%**	**100%**	**0.93**	1	0.89	*Frontal_Mid_Orb_L*
	2-back	SVM(polynomial/rbf)	50	72.7% ± 0.456	75%	71.43%	0.66	1	0.44	*Frontal_Inf_Orb_R* *Postcentral_L* *Frontal_Mid_L*
	1-back	SVM(Linear)	5	68.2% ± 0.477	62.5%	71.43%	0.58	2	0.33	*Cingulum_Post_R* *Cerebellum_6_L*
Betweenness centrality	3-back	KNN	60	81.8% ± 0.395	75%	85.71%	0.75	3	0.60	*Occipital_Sup_L* *Fusiform_R* *Putamen_R*
	2-back	Decision Tree	50	81.8% ± 0.395	87.5%	78.57%	0.77	2	0.62	*Frontal_Mid_L* *Postcentral_L*
	1-back	Decision Tree	50	**95.5% ± 0.213**	**100%**	**92.86%**	**0.94**	2	0.90	*Cerebellum_3_R* *Cerebellum_7b_L*

**Fig. 4:**
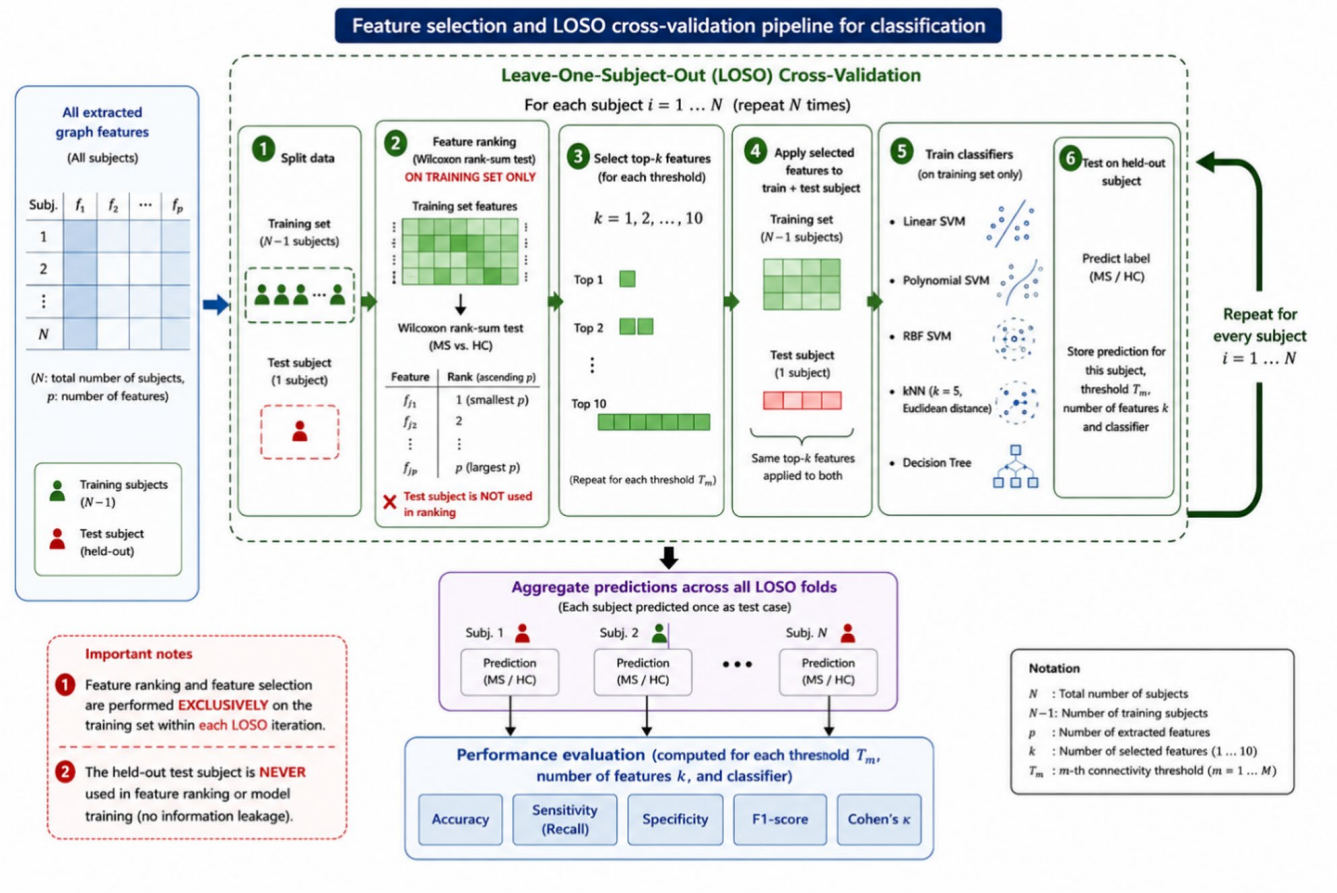
Schematic illustration of the nested cross-validation and feature selection pipeline used for classification

Notably, the same result was also achieved for the betweenness centrality feature in the 1-back network. Overall, the left amygdala, left postcentral, left temporal superior, some parts of the cerebellar region, left orbit middle frontal, right heschel, and right cuneus, which are shown in Fig. 4, were the brain regions that distinguished MS from HC during these different cognitive loads.

It is noticeable that except for k-coreness centrality, the results stem from the 3-back networks obtained in low thresholds. It can display that in a high cognitive load, the smallest number of connections contain discriminative information. While in betweenness centrality the 1-back network created an optimal f1-score of 0.94 by considering 50% of the strongest connections.

**Fig. 5:**
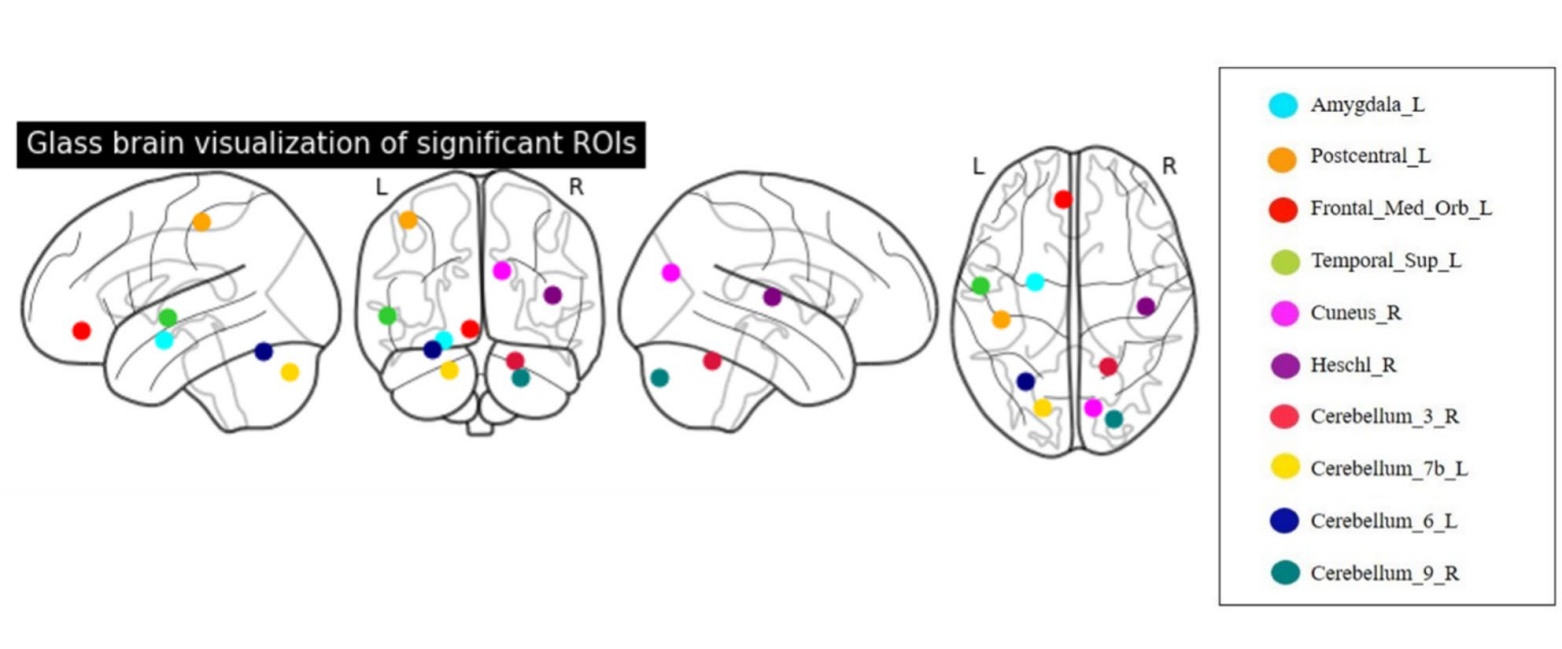
Glass brain visualization of discriminative brain regions

Figure 5 illustrates classification metrics for different cognitive loads. Accordingly, the 3-back network in all local features except betweenness centrality achieved a sensitivity of more than 80% which shows the ability of this network in diagnosing MS patients correctly. What is more, it seems as if by increasing cognitive load the ability of the k-coreness measure in discriminating between two groups became better. The bar graph of specificity shows that the 3-back network in all extracted local features obtained a specificity of more than 80%. However, the betweenness centrality measure extracted from the 1-back network reported a better specificity. It appears that in a higher cognitive load betweenness centrality has been less effective in separating MS patients correctly.

To assess statistical significance, we performed a permutation test with 1000 label shuffles. The observed classification accuracy (95.5%) was significantly higher than chance level (*p* < 0.001). The standard deviation values reported alongside the accuracy metrics were calculated from the subject-level prediction outcomes obtained across the leave-one-subject-out evaluation (i.e., correct vs. incorrect prediction for each held-out subject). Therefore, they reflect the variability of subject-level prediction outcomes and should not be interpreted as variability across repeated cross-validation runs or permutation iterations.

Table 3 summarizes the uncorrected p-values from the regional analyses and highlights regions with comparatively lower values across conditions. Regions 91 to 108, corresponding to cerebellar areas, were among the regions with comparatively lower uncorrected p-values in comparisons between healthy controls and MS patients. Region 101, corresponding to the left cerebellum, was observed across both the 1-back and 2-back networks for all graph measures. Region 9, corresponding to the left middle orbital frontal region, also showed comparatively lower uncorrected p-values in the 3-back network. Because no correction for multiple comparisons was applied, these findings are exploratory and should not be interpreted as definitive regional effects.

**Table 3:** Uncorrected p-values for the examined graph measures and corresponding ROIs

Graph measures	n-back network	PTh (%)	*p*-value	ROI
Clustering coefficient	3-back	40	0.0153	**41**
	2-back	20	0.0390, 0.0464, 0.0464, 0.0464,	**101, 31, 84, 115**
	1-back	55	0.0153, 0.0464	**81, 16**
Page-rank centrality	3-back	35	0.0034, 0.0066, 0.0153, 0.0390	**9, 80, 32, 25**
	2-back	50	0.0034, 0.0390, 0.0390, 0.0464	**57, 105, 116, 38**
	1-back	5	0.0153, 0.0326, 0.0326, 0.0464	**99, 36, 101, 9**
k-coreness centrality	3-back	75	0.0037, 0.0098, 0.0459	**80, 46, 111**
	2-back	40	0.0213, 0.0213, 0.0216, 0.0405	**18, 72, 21, 82**
	1-back	50	0.0014, 0.0014, 0.0014, 0.0014, 0.0014, 0.0171, 0.0186, 0.0232, 0.0339, 0.0369, 0.0369, 0.0371, 0.0371, 0.0371, 0.0371, 0.0371, 0.0371, 0.0371, 0.0397, 0.0397, 0.0397, 0.0397, 0.0397, 0.0397, 0.0397, 0.0399, 0.0399, 0.0479	**20, 21, 63, 69, 92, 81, 102, 72, 57, 114, 115, 1, 13, 15, 19, 70, 71, 75, 44, 91, 93, 94, 101, 104, 108, 34, 73, 11**
Eigenvector centrality	3-back	30	0.0034, 0.0225, 0.0272, 0.326	**9, 80, 25, 28**
	2-back	45	0.0186, 0.0464, 0.0464	**16, 21, 70**
	1-back	60	0.0186, 0.0225, 0.0326	**21, 63, 81**
Degree	3-back	35	0.0011, 0.0081, 0.0219, 0.0323	**9, 80, 32, 25**
	2-back	50	0.0218, 0.0418, 0.0422	**57, 101, 116**
	1-back	5	0.0160, 0.0171, 0.0195	**36, 101, 99**
Betweenness centrality	3-back	60	0.0042, 0.0082, 0.0125, 0.0153, 0.0186, 0.0186, 0.0272, 0.0326, 0.0390, 0.0464	**8, 115, 28, 27, 49, 56, 103, 46, 96, 41**
	2-back	50	0.0125, 0.0186, 0.0326, 0.0390, 0.0390	**7, 57, 101, 9, 67**
	1-back	50	0.0053, 0.0053, 0.0225	**96, 101, 56**

**Fig. 6:**
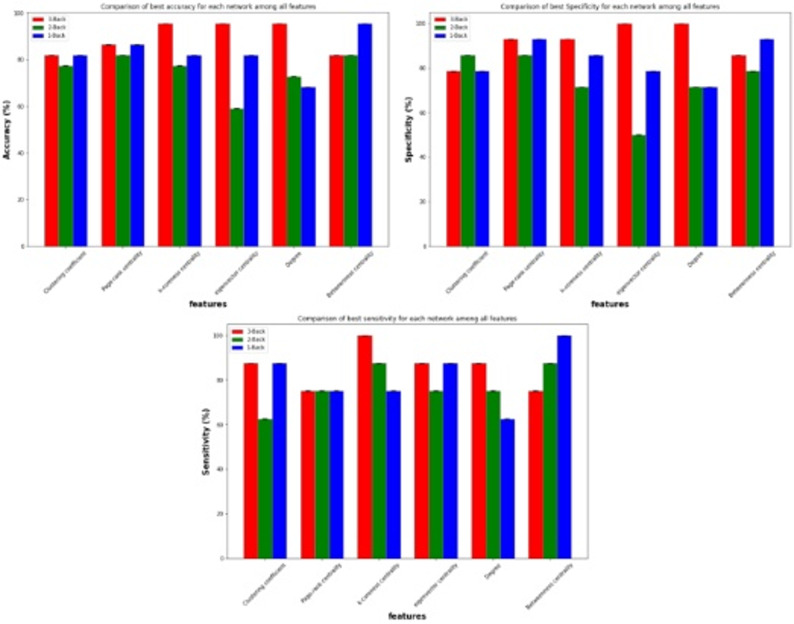
Comparison different cognitive loads based on obtained results from local features

**Fig. 7:**
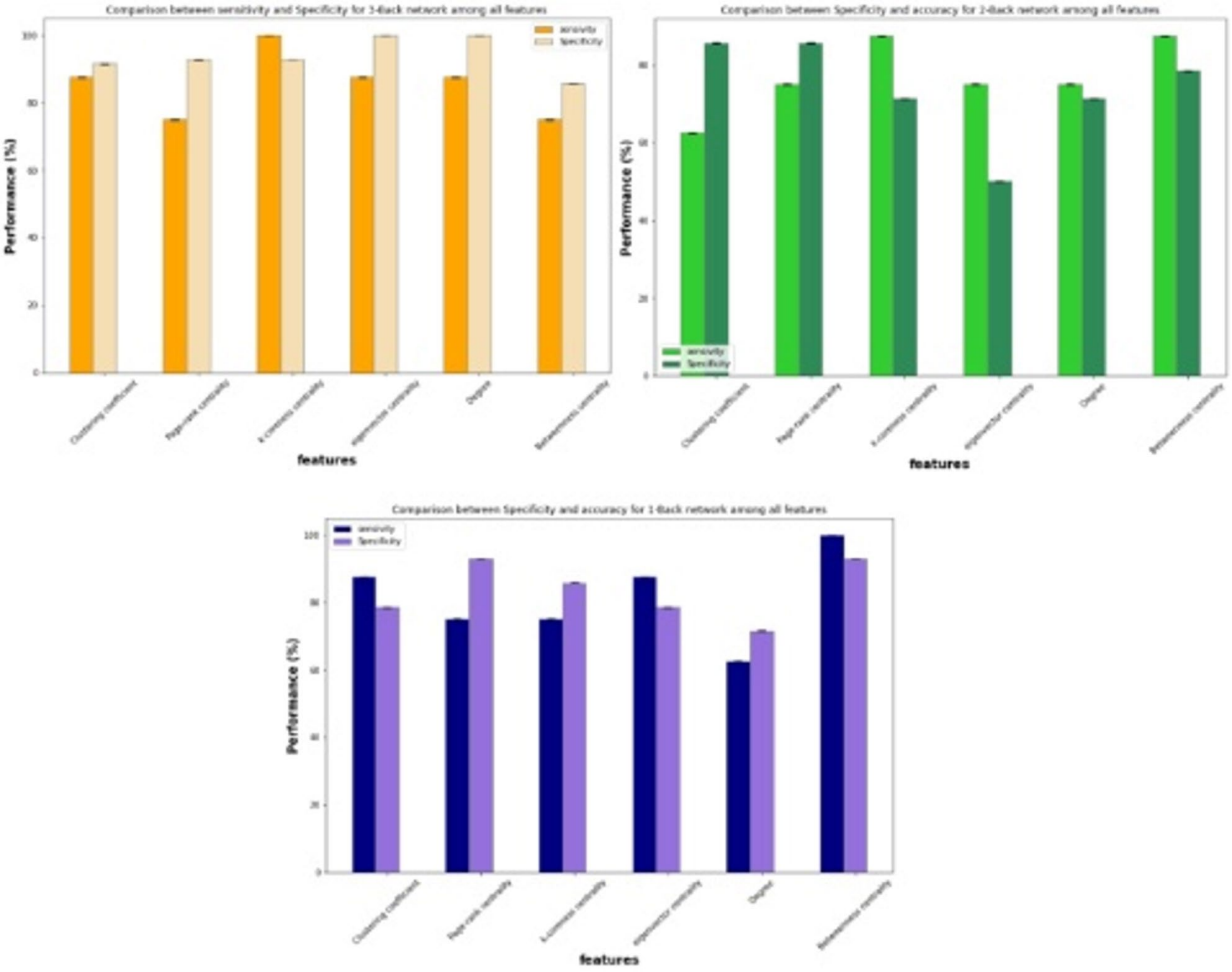
Comparison between sensitivity and specificity for different n-back conditions

Figure 6 depicts a comparison of sensitivity and specificity for different cognitive loads. Extracted features from the 3-back network performed better than the other two networks in classifying MS patients (Fig. 7).

In high cognitive load condition, MS patients were disabled to perform the task to perfection which might stem from poorer functional connectivity in MS patients.

The k-coreness centrality in the 2-back and 3-back networks acquired a sensitivity of more than 80%. In addition, the extracted betweenness centrality measure from the 2-back and 1-back networks displayed specificity and sensitivity of more than 80%. While the clustering coefficient measure which was extracted from the 3-back network and the 1-back network result in a sensitivity of more than 80%.

## Discussion

Working memory deficits are commonly reported in individuals with multiple sclerosis (MS) and may manifest as difficulties in maintaining and manipulating information in real time. Such impairments can influence attention, problem-solving, and decision-making in daily functioning. In the present study, we investigated whether graph-theoretical features of functional brain networks during the n-back task could distinguish MS patients from healthy controls using different machine-learning classifiers.

The main finding of this study was that centrality-based network features derived from the 3-back condition achieved the highest classification performance, reaching an accuracy of 95.5%, which was confirmed to be statistically significant by permutation testing (p < 0.001). This result suggests that brain network organization under high cognitive load provides more discriminative information for classification in the present dataset. Although SVM and decision tree classifiers frequently achieved high performance, comparable accuracies were also observed for kNN in specific feature configurations, indicating that the discriminative power primarily arises from the extracted network features rather than a single classifier.

The n-back paradigm revealed variability across cognitive loads, with the 3-back networks providing the most informative classification features for distinguishing MS patients from healthy controls. Interestingly, some model-selected features were also identified in the 1-back condition, particularly within cerebellar areas, whereas the 2-back condition showed fewer selected features. This pattern differs from studies reporting monotonic increases in functional connectivity with working-memory load and may reflect differences in experimental paradigms or inter-individual variability in cognitive resource recruitment. Previous work in healthy individuals has also suggested that although 2-back and 3-back activation maps may not differ significantly, their functional connectivity patterns can diverge, supporting the load-dependent network reconfiguration observed here [46].

Several features retained by the classification framework originated from regions —including the cerebellum, orbitofrontal cortex, and cingulate-related networks—that have previously been associated with cognitive and executive dysfunction in MS. Functional neuroimaging studies indicate that MS-related structural and functional damage can disrupt the efficient coordination of neural resources required for working memory processes [47, 48]. Moreover, functional connectivity alterations in MS have been reported in both supratentorial regions and the cerebellum, with associations to cognitive performance, while early-stage connectivity changes may reflect compensatory mechanisms that eventually give way to network collapse as pathology progresses [49]. The selection of features associated with cerebellar and orbitofrontal regions in the present results is broadly consistent with prior evidence, although their role here should be interpreted as relative importance within a classification framework rather than definitive region-wise statistical confirmation.

Methodologically, the choice of atlas and graph construction strategy can influence functional connectivity analyses. Although anatomical atlases such as AAL are widely used and facilitate comparability across studies, they may not perfectly correspond to functional boundaries. Future work could benefit from exploring functional atlases, weighted or directed graphs, and effective connectivity approaches to better capture causal and dynamic interactions between brain regions.

Several limitations should be acknowledged. The small and imbalanced sample size (8 MS patients and 14 healthy controls) may limit the stability and generalizability of the findings and increases the susceptibility to overfitting in machine-learning analyses. Although cross-validation and F1-score reporting were used to partially mitigate imbalance effects, the reported performance primarily reflects discriminative capability within this dataset rather than definitive clinical applicability. In addition, the absence of strict correction for multiple comparisons during feature selection may increase the risk of false-positive findings, although embedding feature selection within cross-validation partially reduces this concern. Validation in larger, balanced, and independent cohorts with stronger statistical control will therefore be essential.

Furthermore, given the high dimensionality of the feature space relative to the sample size, the feature selection process may be unstable, and the specific features selected could vary across different samples. Therefore, the identified features should be regarded as exploratory and require confirmation in larger, independent datasets.

In conclusion, the present findings indicate that graph-theoretical centrality features of functional brain networks—particularly under high working-memory load—provide meaningful discriminative information for identifying MS-related cognitive alterations. While preliminary, these results highlight the potential of network-based machine-learning approaches for improving the understanding and assessment of cognitive dysfunction in MS and motivate further validation in larger clinical datasets.

## Conclusions

In conclusion, graph-theoretical features derived from functional brain networks during high cognitive load provided discriminative information for distinguishing people with MS from healthy controls. In such demanding conditions, the observed graph-theoretical features in individuals with MS may be consistent with alterations in functional network integration; however, the selected features should be interpreted as contributors to classification performance rather than confirmed neural markers. Although these findings should be interpreted with caution due to the limited and imbalanced sample size, they underscore the potential value of network-based analytical approaches for improving the understanding and detection of cognitive alterations in MS.

## Data Availability

"The data can be accessed through reasonable request from corresponding author."
